# Mitochondrial morphology is altered in atrophied skeletal muscle of aged mice

**DOI:** 10.18632/oncotarget.4235

**Published:** 2015-05-22

**Authors:** Jean-Philippe Leduc-Gaudet, Martin Picard, Félix St-Jean Pelletier, Nicolas Sgarioto, Marie-Joëlle Auger, Joanne Vallée, Richard Robitaille, David H. St-Pierre, Gilles Gouspillou

**Affiliations:** ^1^ Département des Sciences de l'Activité Physique, Faculté des Sciences, UQAM, Montréal, Canada; ^2^ Groupe de Recherche en Activité Physique Adaptée, Montréal, Canada; ^3^ Centre de Recherche du CHU Sainte-Justine, Montréal, Canada; ^4^ The Center for Mitochondrial and Epigenomic Medicine, Children's Hospital of Philadelphia and University of Pennsylvania, Philadelphia, PA, USA; ^5^ Département de Neurosciences, Université de Montréal, Montréal, Canada; ^6^ Groupe de Recherche sur le Système Nerveux Central, Montréal, Canada; ^7^ Centre de Recherche de l'Institut Universitaire de Gériatrie de Montréal, Montréal, Canada

**Keywords:** mitochondria, muscle aging, atrophy, sarcopenia, mitochondrial dynamics

## Abstract

Skeletal muscle aging is associated with a progressive decline in muscle mass and strength, a process termed sarcopenia. Evidence suggests that accumulation of mitochondrial dysfunction plays a causal role in sarcopenia, which could be triggered by impaired mitophagy. Mitochondrial function, mitophagy and mitochondrial morphology are interconnected aspects of mitochondrial biology, and may coordinately be altered with aging. However, mitochondrial morphology has remained challenging to characterize in muscle, and whether sarcopenia is associated with abnormal mitochondrial morphology remains unknown. Therefore, we assessed the morphology of SubSarcolemmal (SS) and InterMyoFibrillar (IMF) mitochondria in skeletal muscle of young (8-12wk-old) and old (88-96wk-old) mice using a quantitative 2-dimensional transmission electron microscopy approach. We show that sarcopenia is associated with larger and less circular SS mitochondria. Likewise, aged IMF mitochondria were longer and more branched, suggesting increased fusion and/or decreased fission. Accordingly, although no difference in the content of proteins regulating mitochondrial dynamics (Mfn1, Mfn2, Opa1 and Drp1) was observed, a mitochondrial fusion index (Mfn2-to-Drp1 ratio) was significantly increased in aged muscles. Our results reveal that sarcopenia is associated with complex changes in mitochondrial morphology that could interfere with mitochondrial function and mitophagy, and thus contribute to aging-related accumulation of mitochondrial dysfunction and sarcopenia.

## INTRODUCTION

Skeletal muscle aging is characterized by progressive loss of muscle mass and function, a physiological process termed sarcopenia [1]. Although sarcopenia is a complex and multifactorial process, strong evidence indicates that accumulation of mitochondrial dysfunction plays an important role in the skeletal muscle aging process. Indeed, aged skeletal muscle display impaired mitochondrial energetics [2-6] and increased mitochondrial-mediated apoptosis [7-14]. On the other hand, the overexpression of PGC-1α [15], the master regulator of mitochondrial biogenesis, and the overexpression of a mitochondrial-targeted antioxidant enzyme catalase [16] have been shown to attenuate the effect of aging on skeletal muscle. Similarly, the two most efficient non-pharmacological strategies to attenuate sarcopenia, calorie-restriction [17-19] and endurance training [20, 21], are well-known to improve mitochondrial function [22]. Collectively, these findings indicate that sarcopenia involves alterations in multiple aspects of mitochondrial function.

Our understanding of the processes impacting mitochondrial function rapidly evolved over the past decades. For instance, it is now well established that mitochondrial quality control by mitophagy [23], which occurs in parallel with morphology regulation via mitochondrial fusion and fission [24, 25], is critical for the maintenance of normal mitochondrial function. Mitophagy consists of the selective removal of dysfunctional mitochondria, a process regulated by the Parkin-Pink1 pathway [23]. Recent evidence suggests that reduced mitophagy may cause the accumulation of mitochondrial dysfunctions in aging skeletal muscle [7, 26]. In support of this argument, it was recently shown that the genetic impairment of global autophagy, and therefore mitophagy, leads to mitochondrial dysfunction and myofiber atrophy [27]. In contrast, the activation of mitophagy by Parkin overexpression in Drosophila skeletal muscle increases mitochondrial citrate synthase enzymatic activity and attenuates the accumulation of protein aggregates, a marker of cellular aging [28].

Essential to mitophagy is the segregation of segments of the mitochondrial network through mitochondrial fission [25]. This involves dynamic shape changes from elongated filamentous mitochondria, to shorter spheroid organelles of manageable size for autophagic digestion [25]. The converse process where mitochondria can also fuse with one another leads to increase mitochondrial length and branching complexity [29]. Importantly, beyond enabling quality control processes, mitochondrial dynamics and morphology transitions also directly impact several aspects of mitochondrial function that are relevant to skeletal muscle aging. Indeed, mitochondrial morphology and function are interlinked, with changes in mitochondrial morphology affecting mitochondrial function [30-34] and vice versa [35]. Collectively, the available literature in cell culture models establishes that elongated mitochondria most commonly display improved mitochondrial function, including an increase in respiration, a decrease in reactive oxygen species (ROS) production and a decrease in the sensitivity of opening of the apoptosis-regulator permeability transition pore (PTP) [30-32]. In contrast, fragmented mitochondria show a host of impaired function, such as decreased respiration, increased ROS production and sensitisation of the PTP [30, 33, 34, 36].

Well-supported candidate mechanisms for sarcopenia include alterations in mitochondrial function and impaired mitophagy. Given the interrelations between these features and the control of mitochondrial morphology through fusion/fission, it is logical to hypothesize that mitochondrial morphology should be impaired with muscle aging. However, to date, the question of whether mitochondrial morphology is altered during aging-related skeletal muscle atrophy is largely unresolved. To address this issue, we investigated the morphology of sub-sarcolemmal (SS) and Intermyofibrillar (IMF) mitochondria in the white gastrocnemius muscle (known to display significant atrophy with aging) of young and old mice using a recently developed 2-dimensional transmission electron microscopy approach [37, 38]. The contents of pro-fusion and pro-fission proteins involved in mitochondrial dynamics were also investigated. Our results provide quantitative information demonstrating significant mitochondrial morphological alterations in skeletal muscle undergoing aging-related atrophy.

## RESULTS

### Animal characteristics and evidence for aging-related atrophy of the white gastrocnemius muscle

**Figure 1:**
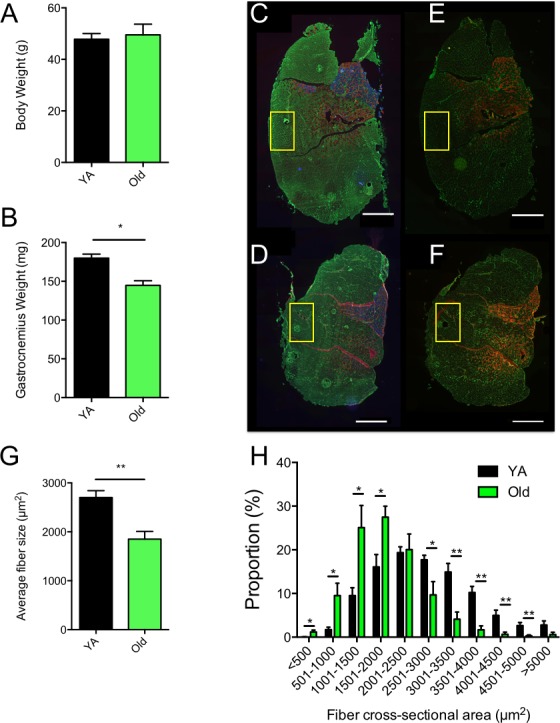
Evidence for atrophy in the white gastrocnemius of aged mice **A.** Whole body weights and **B.** whole gastrocnemius weights of young adult (YA) and Old mice. **C.** and **D.** Skeletal muscle cross-sections of YA (C) and Old (D) Plantaris, soleus and gastrocnemius complexes immunolabeled for type I (blue), type IIa (red) and type IIb (green) myosin heady chains (MHCs). **E.** and **F.** Skeletal muscle cross-sections of YA (E) and Old (F) Plantaris, soleus and gastrocnemius complexes immunolabeled for type IIa (red) and type IIx (green) myosin heady chains (MHCs). In C to F, laminin was stained in green to visualize fiber borders. Yellow squares in C to F correspond to the regions that were processed, on the contralateral White Gastrocnemius (WG), for all TEM analyses detailed in the present manuscript. **G.** average fiber size of the WG of YA and old mice, **H.** Fiber size distribution in the WG of YA and old mice. Scale bars: 1000μm. Data in graphs are presented as Mean ± SEM. *N* = 6 per group. *: *p* < 0.05; **: *p* < 0.01

No difference in body weight was observed between young adult and old mice (Figure 1A). The whole Gastrocnemius (Gas) weight was significantly lower in aged animals (Figure 1B). Old mice also displayed a significant reduction in the mean fiber size (Figure 1C-1G), as well as a significant leftward shift of the fiber size distribution (i.e. increase in the proportion of small fibers) in the White Gas (W. Gas, Figure 1H). No change in fiber type proportion was observed in the W. Gas (in %, Old: 98.6 ± 1.7 type IIb and 1.4 ± 1.7 type IIx; Young adult: 98.5 ± 1.2 type IIb and 1.5 ±1.2 type IIx; *N* = 6). Altogether, our data clearly indicate that the W. Gas of our aged animals exhibited sarcopenia.

### Effects of skeletal muscle aging on mitochondrial content

**Figure 2:**
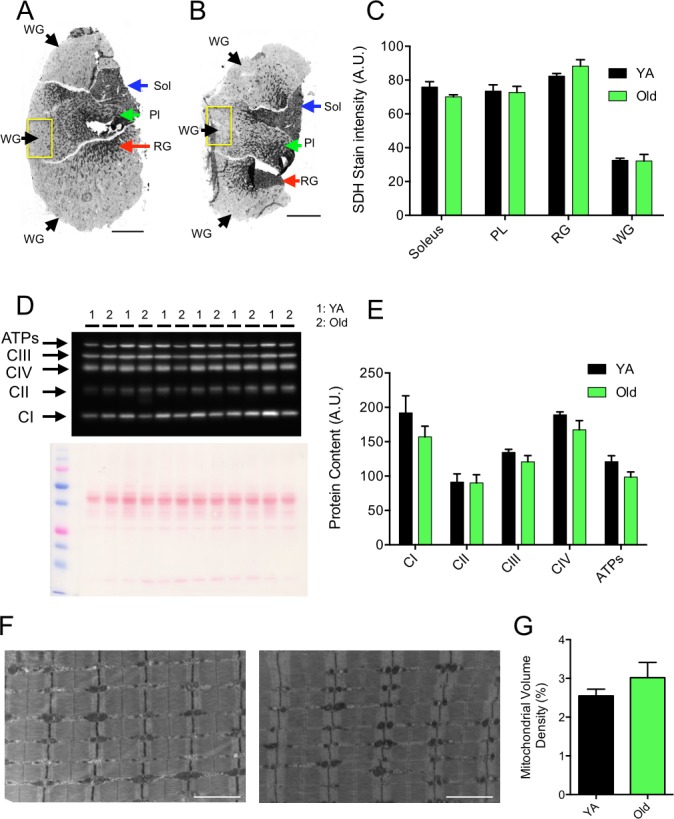
Effects of aging on skeletal muscle mitochondrial content **A.** and **B.** Succinate dehydrogenase (SDH) stain of cross-sections of Young Adult (YA) (A) and Old (B) Plantaris (PL), soleus and gastrocnemius muscles. Yellow squares in A and B correspond to the regions that were processed, on the contralateral white gastrocnemius (WG), for all TEM analyses detailed in the present manuscript. Black, green, red and blue arrows in A and B point toward regions that were used for the quantification of the SDH stain intensity in the WG, PL, red gastrocnemius (RG) and soleus, respectively. **C.** Quantification of the SDH stain intensity in the Soleus, PL, RG and WG of YA and old animals (*N* = 6 in each group). **D.** Immumoblot for representative subunits of complexes I, II, III, IV, and ATP synthase obtained in YA and old WG and the corresponding ponceau stain used to normalize quantifications. **E.** Quantifications of the contents of representative subunits of complexes I, II, III, IV, and ATP synthase in YA and old WG (*N* = 6 in each group). **F.** Representative longitudinal TEM images of YA (left) and Old (right) WG that were used for the quantification of mitochondrial volume density. The results of these quantifications are presented in **G.** (*N* = 4 in each group). Data in graphs are presented as Mean ± SEM. Scale bars in A and B: 1000μm. Scale bars in F: 2μm.

Mitochondrial content was assessed using three complementary approaches. First, skeletal muscle cross-sections of the Gastrocnemius-Plantaris-Soleus complex were stained for succinate dehydrogenase (SDH, complex II of the mitochondrial electron transfer system) activity (Figure 2A to 2C), an approach commonly used to investigate mitochondrial content [7]. No difference in the SDH stain intensity was observed between young and old animals in the W. Gas, Red Gas, Plantaris and Soleus muscles (Figure 2C). Mitochondrial content was also estimated by quantifying the content of representative subunits of key proteins involved in mitochondrial oxidative phosphorylation using western blots (Figure 2D). As can be seen in Figure 2E, no significant difference in the content of representative subunits of complexes I, II, III, IV and the ATP synthase was observed between young and old animals. Finally, mitochondrial volume density was quantified using transmission electron microscopy (TEM) images of the W. Gas in longitudinal orientation (Figure 2F). No change in mitochondrial volume density was observed between young and old mice (Figure 2G). Taken altogether, our results demonstrate that mitochondrial content in the W. Gas was similar between young and old mice.

### Effects of skeletal muscle aging on mitochondrial morphology

**Figure 3:**
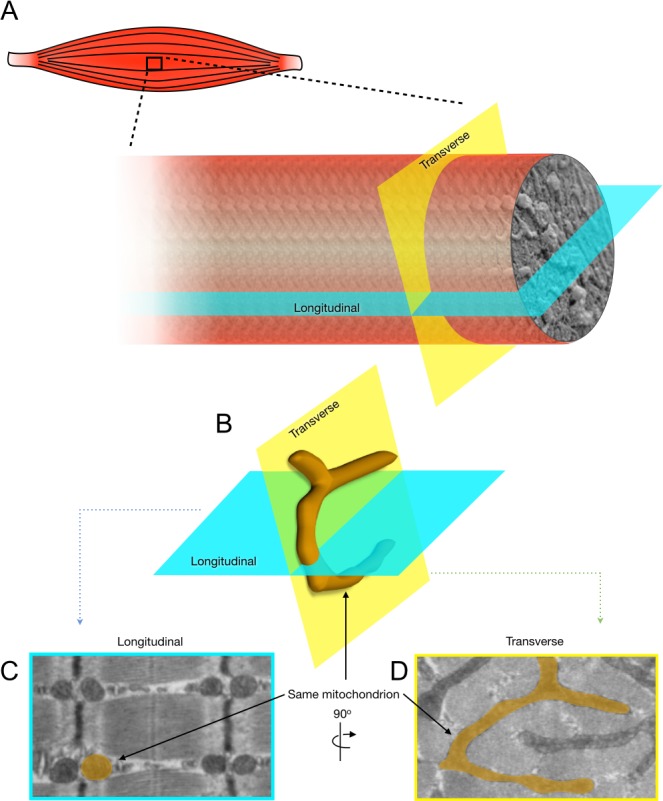
2-Dimensional analysis of mitochondrial morphology in skeletal muscle The present figure illustrates the approach used in the present manuscript to accurately assess the impact of skeletal muscle aging on mitochondrial morphology. **A.** Representation of the longitudinal and transverse orientation of a skeletal muscle fiber. **B.** 3-D representation of a skeletal muscle mitochondrion (highlighted in orange) and its corresponding shapes in longitudinal **C.** and transverse **D.** planes when imaged using transmission electron microscopy.

**Figure 4:**
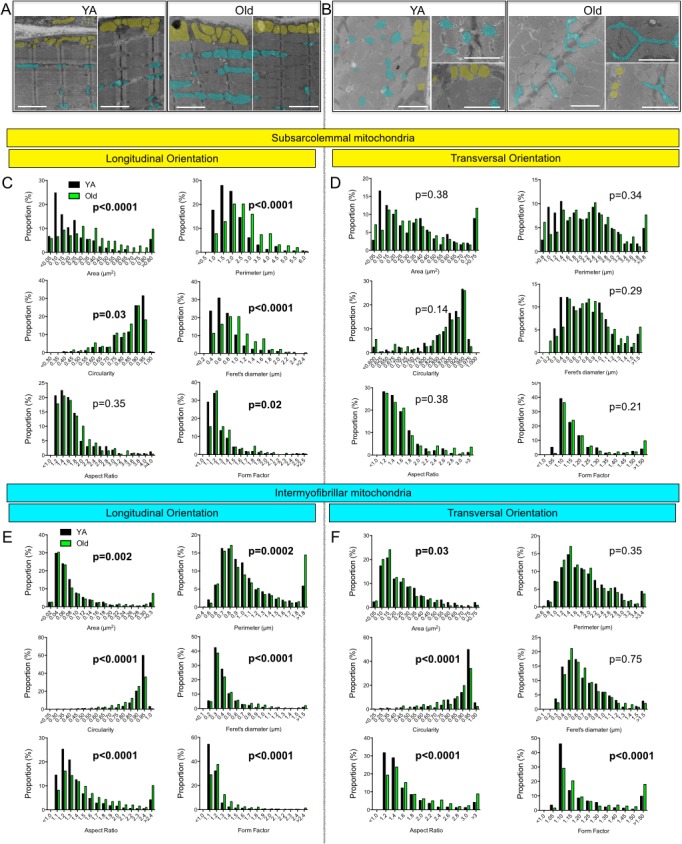
Effects of aging on the morphology of skeletal muscle subsarcolemmal (SS) and intermyofibrillar (IMF) mitochondria **A.** Representative TEM images of the white gastrocnemius (W. Gas.) in longitudinal (A) and transversal **B.** orientations of young adult (YA) and old mice. For clarity, several SS and IMF mitochondria were highlighted in yellow and blue, respectively. **C.** and **D.** Frequency distribution of morphological and shape descriptors for SS mitochondria in longitudinal (C) and transversal orientations (D). **E.** and **F.** Frequency distribution of morphological and shape descriptors for IMF mitochondria in longitudinal (E) and transversal orientation (F). Differences in frequency distributions were tested using a Kolmogorov-Smirnov test comparing cumulative distributions. Scale bars: 2 μm.

To accurately define the impact of aging on mitochondrial morphology, shape descriptors for IMF and SS mitochondria were determined from TEM images acquired in both longitudinal and transversal orientations (Figures 3 and 4).

### Effects of skeletal muscle aging on the morphology of SS mitochondria

When analysed in longitudinal orientation, old skeletal muscles contained SS mitochondria with significantly higher average area, perimeter and Feret's diameter (Table 1). In addition, the distributions of area, perimeter and circularity of SS mitochondria were shifted to the left, while the distribution of form factor was shifted to the right in aged skeletal muscle in longitudinal orientation (Figure 4C), indicating fewer round mitochondria, and a greater proportion with more complex shapes. In transverse orientation, with the exception of a higher average perimeter in old SS mitochondria, none of the shapes descriptors quantified (average and distribution) differed for SS mitochondria (Figure 4D). Taken altogether, these results indicate that aged muscles display a higher proportion of enlarged and less circular SS mitochondria.

**Table 1:** Effects of skeletal muscle aging on morphometric amd shape descriptors of subsarcolemmal and intermyofibrillar mitochondria

	SS		IMF	
	YA	Old	YA	Old
**Longitudinal** **Orientation**				
Area (μm^2^)	0,23 ± 0,24	0,39 ± 0,29*	0,09 ± 0,16	0,11± 0,15
Perimeter (μm)	1,76 ± 0,91	2,37 ± 1,07**	1,02 ± 0,58	1,17 ± 0,78**
Circularity	0,83 ± 0,11	0,80 ± 0,13	0,87 ± 0,08	0,83 ± 0,12*
Feret's Diameter (μm)	0,66 ± 0,38	0,88 ± 0,42**	0,37 ± 0,23	0,44 ± 0,33**
Aspect Ratio	1,65 ± 0,66	1,67 ± 0,57	1,38 ± 0,48	1,63 ± 0,81**
Form Factor	1,24 ± 0,24	1,29 ± 0,27	1,143 ± 0,16	1,22 ± 0,29**
N	165	258	1027	1309
**Transverse** **Orientation**				
Area (μm^2^)	0,34 ± 0,29	0,38 ± 0,34	0,24 ± 0,18	0,21 ± 0,18
Perimeter (μm)	2,05 ± 0,94	2,23 ± 1,12**	1,85 ± 0,83	1,76 ± 0,78**
Circularity	0,87 ± 0,09	0,85 ± 0,13	0,85 ± 0,14	0,81 ± 0,15
Feret's Diameter (μm)	0,75 ± 0,35	0,82 ± 0,44	0,67 ± 0,33	0,66 ± 0,30
Aspect Ratio	1,47 ± 0,44	1,59 ± 0,67	1,55 ± 0,75	1,79 ± 0,82**
Form Factor	1,16 ± 0,15	1,23 ± 0,35	1,23 ± 0,39	1,31 ± 0,38**
N	248	196	387	442

Mean ± SD. Data were obtained from 4 different animals in each group. Ordinary one –way ANOVA with Sidak's multiple comparison test. YA = young adult

* p<0.05;

** p<0.01 vs YA.

### Effects of skeletal muscle aging on the morphology of IMF mitochondria

IMF mitochondria are positioned between myofibrils and exhibit more complex shapes and tri-dimensional spatial distributions than SS mitochondria [38]. In longitudinal orientation, IMF mitochondria of aged mice W. Gas displayed a significant increase in their average perimeter, form factor, aspect ratio and Feret's diameter, as well as a significant decrease in their average circularity (Table 1). In this orientation, no change in their average area was observed (Table 1). Interestingly, IMF mitochondria showed the most significant age-related differences in the distributions of all shape descriptors (Figure 4E). Indeed, the distribution of area, perimeter, Feret's diameter, aspect ratio and form factor of IMF mitochondria were all shifted to the right in aged skeletal muscle (Figure 4E), while the distribution of circularity was shifted to the left in old muscles (Figure 4E). This reflects an age-related loss of round/globular mitochondria to the profit of more elongated organelles spanning the sarcomere length.

In transverse orientation, which represents a single sarcomere plane, IMF mitochondria of old skeletal muscle displayed a significant decrease in their average perimeter associated with increases in their average aspect ratio and form factor values (Table 1). The distributions of the area and circularity values were shifted to the left in old skeletal muscle IMF mitochondria, while the distributions of aspect ratio and form factor were shifted to the right (Figure 4F). Taken altogether, these results indicate that aged IMF mitochondria are less circular, are elongated and show increased branching. These results therefore suggest that with aging, IMF mitochondria morphology increases in complexity.

### Effects of skeletal muscle aging on morphological complexity of SS and IMF mitochondria

**Figure 5:**
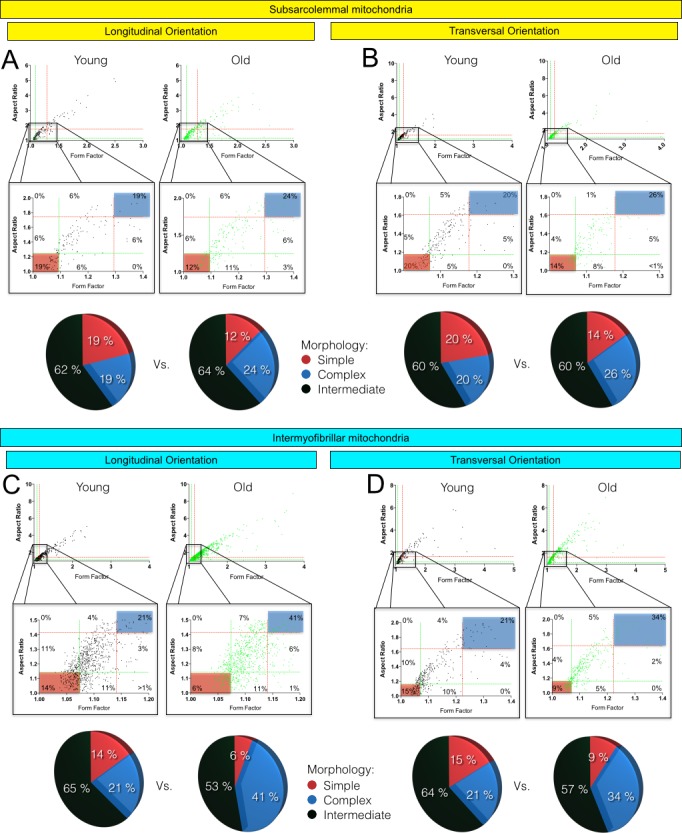
Form factor and aspect ratio distributions for individual subsarcolemmal (A and B) and intermyofibrillar (C and D) mitochondria in longitudinal (A and C) and transversal (B and D) orientations In the first line of A, B, C and D, the values form factor and aspect ratio distributions for individual mitochondria are presented for young adult (left graph) and old (right graph) animals. The second line corresponds to a zoom on the data presented in the first line. In A, B, C and D, green and red dashed lines represent the 25th and 75th percentiles for either aspect ratio or form factor values of young adult, respectively. The pie charts in the third line represent the percentage of mitochondria with simple (i.e. mitochondria with aspect ratio and form factors values inferior to the 25th percentile of young adult values), complex (i.e. mitochondria with aspect ratio and form factors values above the 75th percentile of young adult values) and intermediate (i.e. neither simple nor complex).

To specifically assess the effects of aging on morphological complexity for SS and IMF mitochondria, we plotted the aspect ratio and form factor values for all individual SS and IMF mitochondria (Figure 5). We then determined in young adult and aged animals the proportion of mitochondria that were below the 25^th^ percentile of young adult values for both aspect ratio and form factor (morphologically simple). Similarly, we also determined in young adult and aged animals the proportion of mitochondria that were above the 75^th^ percentile of young adult values for both aspect ratio and form factor (morphologically complex) (Figure 5). As presented in Figure 5A and 5B, only a slight increase in the proportion of SS mitochondria with a complex morphology was observed in aged animals (Figure 5A and 5B). However, the proportion of IMF mitochondria with a complex morphology (branched and elongated) was much higher (close to 1.6 and 2 folds higher in transversal and longitudinal orientations, respectively) in old mice as compared to their young adult littermates (Figure 5C and 5D).

### Effects of skeletal muscle aging on the content of proteins regulating mitochondrial dynamics

**Figure 6:**
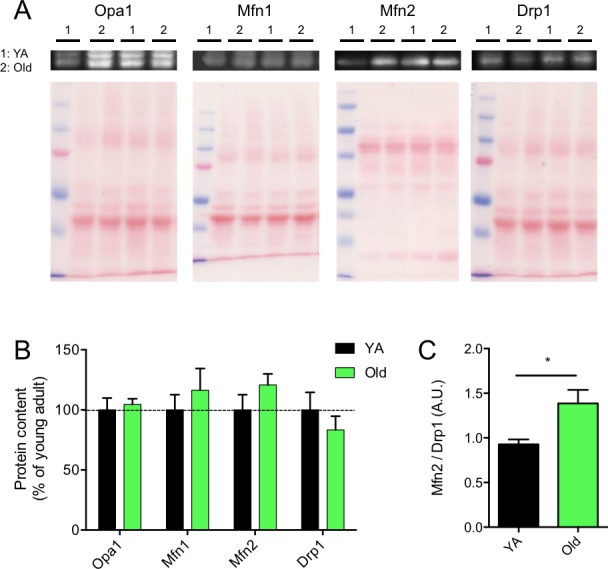
Effects of skeletal muscle aging on the content of proteins regulating mitochondrial dynamics **A.** Representative immunoblots of OPA1, Mfn1, Mfn2 and Drp1 as well as their corresponding ponceau stains. **B.** Quantification of OPA1, Mfn1, Mfn2 and Drp1 protein contents (normalized to their corresponding ponceau stain intensities) in the white gastrocnemius of young adult (YA) and old mice. **C.** Mfn2 to Drp1 protein content ratio. Data in graphs are presented as Mean ± SEM. *N* = 6 per group. *= *p* < 0.05.

To assess the effects of aging on processes involved in mitochondrial dynamics, the contents of key proteins involved in mitochondrial fusion and fission were assessed in young adult and aged skeletal muscles. No significant difference in the content of proteins involved in mitochondrial fusion (Opa-1, Mfn1 and Mfn-2) and fission (Drp1) were observed between young adult and aged W. Gas (Figure 6A and 6B). However, we found that the ratio between Mfn2 and Drp1, an index of the balance between fusion and fission processes, was significantly increased in aged skeletal muscles (Figure 6C).

## DISCUSSION

The aim of the present study was to define whether aging-related muscle atrophy was associated with significant changes in mitochondrial morphology. To this end, we used a quantitative 2-dimensional electron microscopy approach to evaluate the morphology of the two populations of skeletal muscle mitochondria, IMF and SS, in the W. Gas muscle. With this innovative design and approach, atrophied aged skeletal muscle displayed larger and less circular SS mitochondria, as well as longer and more branched (more complex) IMF mitochondria (Figures 4 and 5). While no differences were seen in the content of the pro-fusion proteins Opa1, Mfn1 and Mfn2 and the content of the pro-fission protein Drp1, these morphological changes were associated with an increase in the Mfn2-to-Drp1 ratio, suggesting a fusion-fission imbalance in favour of mitochondrial fusion in aged skeletal muscles (Figure 6).

Although the W. Gas has a relatively low mitochondrial content, we believe this skeletal muscle was ideal for the investigation of the effects of muscle aging on mitochondrial morphology. First of all, the W. Gas is well known to undergo significant aging-related atrophy [2, 3] (Figure 1), a critical requirement when attempting to define cellular processes that might be involved in sarcopenia. Second, this muscle is extremely homogeneous in its fiber-type composition, with approximately 98-99% of type IIb fibers and approximately 1-2% of type IIx (Figure 1). This feature is particularly important when considering the fact that mitochondrial function differs between fiber types [39]. Given the strong relationship that exists between mitochondria function and morphology [40], it appears logical to hypothesize that mitochondrial morphology might also differ between fiber types, although no quantitative analysis has been reported. Fiber-type differences could confound any analysis of mitochondrial morphology. With this in mind, it is therefore important to note that no change in fiber type composition was observed between young and old animals in the W. Gas. in the present study (See Results section and Figure 1). As such, we believe that the changes in mitochondrial morphology reported here are not consequences of a change in muscle fiber phenotype with aging but are a true result of the skeletal muscle aging process.

A current view in the field of skeletal muscle aging, resting on limited evidence, suggests a progressive fragmentation of the mitochondrial network [41]. This notion is based on previous TEM studies limited to longitudinal orientation, reporting an aging-related decrease in IMF mitochondrial size [42-44]. Sectioning skeletal muscle in the transverse orientation is however critical to adequately analyse IMF mitochondrial morphology [38]. This is because IMF mitochondria are tubular and branched structures that align along the sarcomeric plane (in transverse orientation) [45], which essentially appear as round structures when visualized in longitudinal orientation [37]. Taking these factors into consideration, the present study is to date the most thorough investigation of mitochondrial morphology in the context of aging, since multiple representative shape descriptors were quantified in longitudinal and transverse orientations for both SS and IMF mitochondria. In addition, the presence of muscle atrophy and absence of fiber type shift in our model reinforces our confidence that aging-related changes in IMF and SS mitochondrial morphologies are more complex than originally thought.

Because strong evidence indicate that mitochondrial morphology and function are linked [37], it is tempting to speculate that the changes in mitochondrial morphology reported in the present study might play an important role in the well-documented alteration of mitochondrial function in sarcopenic muscle [2-13]. Accordingly, highly enlarged and/or highly branched mitochondria display impaired function (such as reduced ATP production capacity) [46, 47]. Our results show that aging differentially impacts IMF and SS mitochondrial morphologies (Table 1 and Figures 4 and 5). Interestingly, muscle aging is known to differentially impact the functioning of SS and IMF mitochondria. Indeed, SS mitochondria are more affected by the aging process, exhibiting greater uncoupling, significant loss in membrane potential and greater increase in their susceptibility to trigger apoptosis as compared to IMF mitochondria [9]. Our results, showing differential aging-related changes in SS and IMF mitochondrial morphologies, could therefore provide a structural explanation for the differential impact of aging on the function of these two mitochondrial populations in skeletal muscles.

The more elongated and complex morphology of aged muscle mitochondria may explain their increased vulnerability to stress. A recent study compared mitochondrial function (respiration, ROS production and apoptotic susceptibility) in skeletal muscles of young and old rats using two different experimental approaches: i) mitochondrial isolation, which disrupts normal morphology, yielding uniformly spherical organelles; and ii) the less disruptive permeabilized myofiber approach which preserves normal mitochondrial architecture [14, 48]. Age-related functional alterations were strikingly exaggerated in isolated mitochondria compared to that of the non-disruptive conditions [14]. Our present results offer a plausible mechanism to explain this susceptibility of aged skeletal muscle mitochondria to isolation stress. Due to the reported increase in branching and length of IMF mitochondria, it is likely that mitochondrial isolation would cause more damage to the architecture of aged mitochondria than to the initially more globular mitochondria of the young muscle, and therefore altering their function to a greater extent as compared to young mitochondria.

An important question arising from our work is what could induce mitochondrial elongation and branching in aged muscle? Mitochondria become tubular and elongate when processes involved in fusion outpace fission events. Interestingly, mitochondrial fusion can occur in response to mild stress [49], and may represent a compensatory mechanism to improve bioenergetics efficiency in response to such stress [31]. In aged muscle, a number of factors suggest the presence of mild bioenergetics stress, including a reduction in mitochondrial affinity for ADP [2] and activation of the energy sensor AMPK [50]. The fact that aged mitochondria become more elongated would indeed suggest that an intrinsic mitochondrial defect pervades in aged muscle, being in part compensated for by increased fusion. This is consistent with our results, demonstrating increased SS mitochondrial size and length, and branching of IMF mitochondria, as well as the increase in the Mfn2 to Drp1 ratio (Figure 6), all indicative of increased fusion and/or decreased fission in sarcopenic muscle. Again, disruption of this mechanism by the mechanical fragmentation of mitochondria during isolation would be expected to cause more profound functional alterations than in young muscle, as previously reported [14].

Amongst the theories proposed to explain the aging-related accumulation of mitochondrial dysfunction and loss of muscle mass, the mitochondrial-lysosomal theory of aging is currently attracting a growing amount of attention [51]. This theory postulates that autophagy, and therefore mitophagy, decreases with aging in long-lived post-mitotic tissues, leading to an accumulation of dysfunctional and enlarged “giant” mitochondria [51, 52]. The significant enlargement of SS mitochondria and the increased branching and length of IMF mitochondria reported in the present manuscript (Figures 4 and 5) is consistent with and further support this theory. Because mitochondrial fusion is important for the maintenance of mitochondrial function and integrity, [33, 53, 54] and given the reduction in mitophagic potential we previously reported [7], it could be that the mitochondrial morphology changes observed here have resulted from an increase in fusion to compensate for impaired mitophagy. However, because mitophagy requires mitochondrial fragmentation through fission, changes in mitochondrial dynamics, leading to the enlarged SS mitochondria and more complex IMF mitochondria we observed, might render mitochondria refractory to mitophagy, therefore accounting for the overall reduction in mitophagy. Further studies are needed to untangle causes from consequences in the aging-related changes in mitophagy, and the now demonstrated alterations in mitochondrial morphology.

In conclusion, the present study shows that skeletal muscle atrophy occurring with aging is associated with complex changes in mitochondrial morphology, SS mitochondria being enlarged and IMF mitochondria displaying an increase in their morphological complexity (increased branching and length). Our results also suggest that these aging-related changes in mitochondrial shapes result from a change in the fusion/fission balance in favour of mitochondrial fusion. Given the emerging role of mitochondrial morphology and dynamics in the regulation of skeletal muscle mass [55-57], the mitochondrial morphology changes demonstrated by our quantitative approach could potentially contribute to sarcopenia. Studies investigating whether modulating mitochondrial dynamics and morphology can impact the course of sarcopenia are now required.

## MATERIALS AND METHODS

### Animals and muscle tissue harvest

All experiments were approved by the Comité institutionnel de protection des animaux de l'UQAM (#1114-883-1115) and the Comité de déontologie animale of Université de Montréal (#14-069). Six young adult (8- to 12-wk-old) and six aged (88-96 wk-old) male mice, fed ad-libitum with standard chow and with unrestricted access to a running wheel, were studied. Animals were anesthetized with isoflurane and subsequently euthanized by cervical dislocation. The gastrocnemius (Gas) from the left leg was removed. The white portion of the Gas (W. Gas) was separated from the rest of the muscle and cut in half. The first half of the W. Gas was sliced into small pieces (>1mm in thickness) and prepared for TEM analyses. The rest was frozen in liquid nitrogen for western blot experiments and stored at −80°C until use. The Gas, Plantaris and Soleus complex from the right leg were removed. A slice through the entire midbelly of this complex was mounted on cork in optimal cutting temperature compound and frozen in liquid isopentane cooled in liquid nitrogen. Histology samples were stored at −80°C until use.

### Transmission electron microscopy

Detailed procedures are available in [37, 38]. Briefly, W. Gas samples were immediately fixed in a 2% glutaraldehyde solution in 0.1 M cacodylate buffer (pH 7.4), postfixed in 1% osmium tetroxide in 0.1 M cacodylate buffer, dehydrated in increasing concentration of ethanol and propylene oxide and embedded in Epon. One μm thick sections were stained with toluidine blue to verify the orientation of the muscle tissue prior the ultrathin sectioning. Ultrathin sections were cut in longitudinal or transverse orientation using an Ultracut S ultramicrotome (Leica) and mounted on nickel carbon-formvar coated grids. Uranyl acetate and lead citrate stained sections were imaged using a Philips CM100 electron microscope (FEI). Digital micrographs were captured using an AMT XR80 CCD digital camera at x7900 magnification.

Individual SS and IMF mitochondria from 4 young adult and 4 old mice were manually traced in longitudinal and transverse orientations using in ImageJ (NIH) and to quantify the following morphological and shape descriptors: area (in μm^2^), perimeter (μm), circularity (4π·(surface area/perimeter2)), Feret's diameter (longest distance (μm) between any two points within a given mitochondrion, aspect ratio ((major axis)/(minor axis)); a measure of the “length to width ratio”) and, form factor ((perimeter)/(4π·surface area); a measure sensitive to the complexity and branching aspect of mitochondria) [37, 38, 58]. Details on the number of SS and IMF mitochondria that were traced in both transverse and longitudinal orientations are available in Table 1.

### Skeletal muscle sample sectioning for histology

Eight micron thick serial cross-sections were cut in a cryostat at −18 °C and mounted on lysine coated slides (Superforst) to determine fiber type and mitochondrial content as described in [59].

### *In situ* determination of fiber type

Two first sections were immunolabeled for the different myosin heavy chains (MHC) as previously described [7, 59]. Briefly, the first cross-sections of each animal sample were used to immunolabel for MHC type I, IIa and IIb. These cross-sections were first allowed to reach room temperature and rehydrated with PBS (pH 7.2). These sections were then blocked using goat serum (10% in PBS) and incubated for 1 hour at room temperature with the following primary antibody cocktail: mouse IgG2b monoclonal anti-MHC type I (BA-F8, 1:25), mouse IgG1 monoclonal anti-MHC type IIa (SC-71, 1:200), mouse IgM monoclonal anti-MHC type IIb (BF-F3, 1:200) and rabbit IgG polyclonal anti-laminin (Sigma L9393, 1:750). Muscle cross-sections were then washed three times in PBS before being incubated for 1 hour at room temperature with the following secondary antibody cocktail: Alexa Fluor 350 IgG2b (y2b) goat anti-mouse (Invitrogen, A-21140, 1:500), Alexa Fluor 594 IgG1 (y1) Goat anti-mouse (Invitrogen, A-21125, 1:100), Alexa Fluor 488 IgM goat anti-mouse (Invitrogen, A-21042, 1:500) and Alexa Fluor 488 IgG goat anti-rabbit (A-11008, 1:500). Muscle cross-sections were then washed three times in PBS and slides were then cover slipped using Prolong Gold (Invitrogen, P36930) as mounting medium.

Identical procedures were employed for the section cross-sections used to immunolabel for MHC type IIx, except the primary antibody cocktail which was comprised of a mouse IgM monoclonal anti-type 2x MHC (6H1, 1:25), a mouse IgG1 monoclonal anti-MHC type IIa (SC-71, 1:200) and a rabbit IgG polyclonal anti-laminin, and the secondary antibody cocktail that was comprised of Alexa Fluor 488 IgM goat anti-mouse, Alexa Fluor 594 IgG1 (y1) Goat anti-mouse (Invitrogen, A-21125, 1:100), and Alexa Fluor 488 IgG goat anti-rabbit. All primary antibodies targeting MHCs were purchased from the Developmental Studies Hybridoma Bank (DSHB, University of Iowa, IA). Note that an average of 634 ± 140 (young adult) and 724 ± 218 (old) fibers were used to assess muscle fiber size and type proportion (Figure 1).

### *In situ* determination of mitochondrial content using the SDH stain

Muscle cross-sections were stained for succinate dehydrogenase (SDH, complex II of the respiratory chain) activity as follows [59]: Sections were first allowed to reach room temperature and were rehydrated with PBS (pH 7.2). Sections were then incubated in a solution containing Nitroblue tetrazolium (1.5 mM), Sodium succinate (130 mM), Phenazine methosulphate (0.2 mM) and Sodium azide (0.1 mM) for 20 min. Cross-sections were then washed 3 times in PBS, dehydrated in 75% (30 s), 90% (30 s) and 100% (10 min) ethanol and cover-slipped using an aqueous mounting medium (Vector Labs, VectaMount AQ Medium, H-5501). All samples were processed at the time and using the same incubation solution, ensuring that all samples underwent the exact same conditions.

### Immunoblotting

Protein contents of OPA1, Mfn1, Mfn2 and Drp1, as well as representative subunits of key proteins involved in mitochondrial oxidative phosphorylation were determined in muscle homogenates prepared from W. Gas muscles. Approximately 10 mg of the each muscle was homogenized in 10 volumes of an extraction buffer composed of Tris base 50 mM, NaCl 150 mM, Triton X-100 1%, Sodium deoxycolate 0.5%, SDS 0.1% and 10μl/ml of a protease inhibitor cocktail (Sigma P8340). The homogenate was centrifuged at 15,000 *g* for 15 min at 4°C. Protein content in the supernatant was determined using the Bradford method.

Aliquots of supernatant were mixed with Laemli buffer and subsequently boiled at 95°C for 5 min. Approx. 30 μg of proteins were loaded onto 8 to 12% gels, electrophoresed by SDS-PAGE and then transferred to polyvinylidene fluoride membranes (Life Sciences). Membranes were incubated for 1 h at room temperature in a blocking buffer composed of 5% (w/v) non-fat dried milk in Tris-buffered saline containing 0.1% Tween 20 (TBS-T) and then incubated for 1h with rabbit anti-OPA1 (Abcam Ab42364; 1:1000), rabbit anti-Mfn1 (Proteintech, 13798-1-AP; 1:500) rabbit Mfn2 (Sigma, M6319; 1:1000), rabbit anti-Drp1 (Cell Signaling, D6C7, 1:1000) and OXPHOS Blot (MitoSciences, MS6034; 1:500) antibodies diluted in blocking buffer. Membranes were washed 6 times for 5 min each in TBS-T and subsequently incubated with HRP-conjugated secondary antibodies (Abcam Ab6721 and Ab6728) diluted in blocking buffer 1 h at room temperature. Signals were detected using enhanced chemiluminescence substrate (Biorad, Clarity ECL substrate, 170-5060) and analyzed using ImageJ (NIH).

### Statistical analyses

Differences in animal masses, muscle masses, fiber size, SDH stain intensity, protein contents, mitochondrial density were tested with unpaired bilateral student t-tests. Differences for average values of shape descriptors used to assess mitochondrial morphology were tested with a Ordinary one-way ANOVA with Sidak's multiple comparison test. Differences in the distribution of shape descriptor values used to assess mitochondrial morphology were tested using a Kolmogorov-Smirnov test comparing cumulative distributions. All statistical analyses were performed using Prim 6 (GraphPad).
